# Supplementary material for: Modeling methyl-sensitive transcription factor motifs with an expanded epigenetic alphabet
Source: Genome Biol. 2024 Jan 8;25:11. doi: 10.1186/s13059-023-03070-0 (PMC10773111; doi:10.1186/s13059-023-03070-0)
Supplement: Supplementary file 1 — Additional file 1: Fig. S1. Stepwise epigenetic modification of cytosine. Fig. S2. Relationship between unmodified versus modified motif statistical significance of central enrichment (from CentriMo [53]) and modified base calling thresholds across different whole genome bisulfite sequencing (WGBS) and oxidative WGBS (oxWGBS) specimens, in mice [48]. Fig. S3. Relationship between unmodified versus modified ZFP57 statistical significance of central enrichment (from CentriMo [53]) and modified base calling thresholds across different WGBS and oxWGBS specimens, in mice [48]. Fig. S4. Relationship between unmodified versus modified C/EBPβ statistical significance of central enrichment (from CentriMo [53]) and modified base calling thresholds across different WGBS and oxWGBS specimens, in mice [48]. Fig. S5. ZFP57 (Strogantsev et al. [20] CB9; 56 142 ChIP-seq peaks) CentriMo analysis of de novo and JASPAR motifs (Methods). Fig. S6. CentriMo [53] results for OCT4 cleavage under targets and release using nuclease (CUT&RUN) in mouse embryonic stem cells (mESCs). Fig. S7. Modified versus unmodified motifs, combining score and cluster information, for a wide array of transcription factors. [file 13059_2023_3070_MOESM1_ESM.pdf]

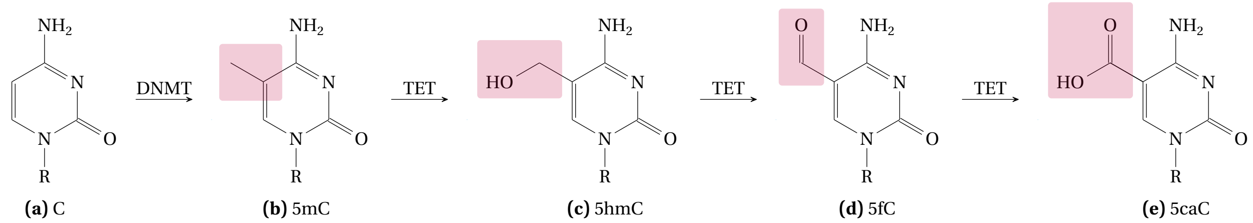

Fig. S1. Stepwise epigenetic modification of cytosine. (a) DNA methyltransferase (DNMT) methylates cytosine to create **(b)** 5-methylcytosine (5mC), which ten-eleven translocation (TET) enzymes oxidize to create **(c)** 5-hydroxymethylcytosine (5hmC), which TET again oxidizes to create **(d)** 5-formylmethylcytosine (5fC). Finally, TET can further oxidize 5fC to **(e)** 5-carboxylmethylcytosine (5caC), which can then return to an unmodified cytosine through decarboxylation or thymine DNA glycosylase (TDG) mediated excision, followed by base excision repair. R indicates deoxyribose and the rest of a DNA molecule. Purple rectangles indicate functional groups changed in the reaction.

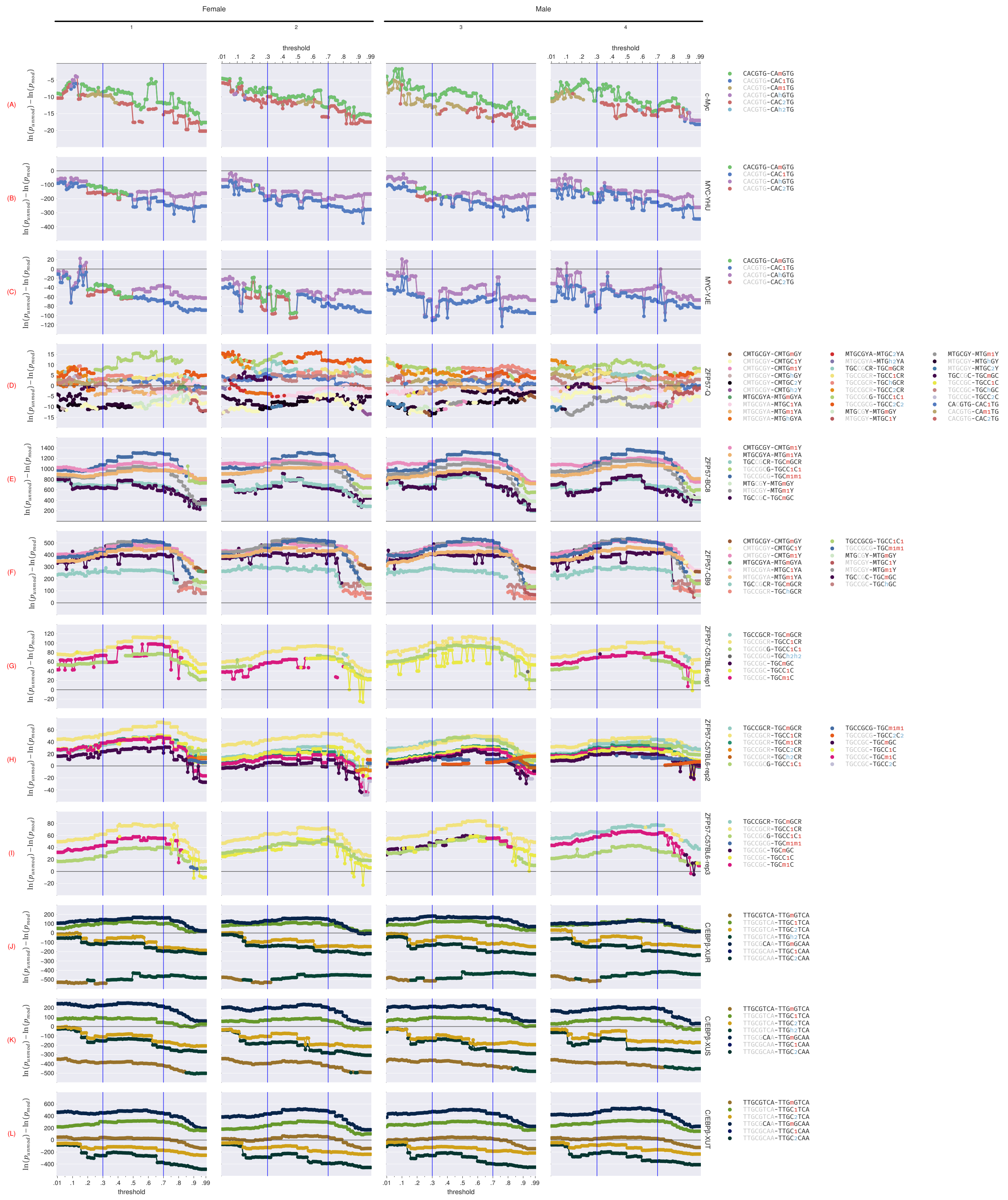
Female
Male
threshold
ln(p_unmod) − ln(p_mod)
c-Myc
MYC-YHU
MYC-YJE
ZFP57-Q
ZFP57-BC8
ZFP57-CB9
ZFP57-C57BL6-rep1
ZFP57-C57BL6-rep2
ZFP57-C57BL6-rep3
C/EBPβ-XUR
C/EBPβ-XUS
C/EBPβ-XUT
(A)
(B)
(C)
(D)
(E)
(F)
(G)
(H)
(I)
(J)
(K)
(L)

Fig. S2 (preceding page). Relationship between unmodified versus modified motif statistical significance of central enrichment (from CentriMo[55]) and modified base calling thresholds across different whole genome bisulfite sequencing (WGBS) and oxidativeWGBS (oxWGBS) specimens, in mice.[51] We compared each unmodified motif, at each threshold, to its top three most significant modifications for c-Myc, C/EBPβ, and replicate 2 of the C57BL/6 ZFP57 samples, but only the single most significant modification for all other ZFP57 samples. The displayed motif pairs changes at individual thresholds, depending on which motif pairs stay in the top three. We called all ZFP57 peaks, excepting Quenneville et al.[22], using a stringent MACS[62] threshold of $q = 0.00001$. Sign of value indicates preference for the unmodified (negative) motif or the modified (positive) motif. Rows: single chromatin immunoprecipitation-sequencing (ChIP-seq) replicates for a particular transcription factor target, consisting of: 3 c-Myc (Krepelova et al.[59], `ENCFF001YHU`, and `ENCFF001YJE`), 6 ZFP57 (from Quenneville et al.[22], and BC8 and CB9 cells, from Strogantsev et al.[23]), and 3 C/EBPβ (`ENCFF001XUR`, `ENCFF001XUS`, and `ENCFF001XUT`). Columns: replicates of WGBS and oxWGBS. We additionally depict C/EBPβ alone in Fig. S4.

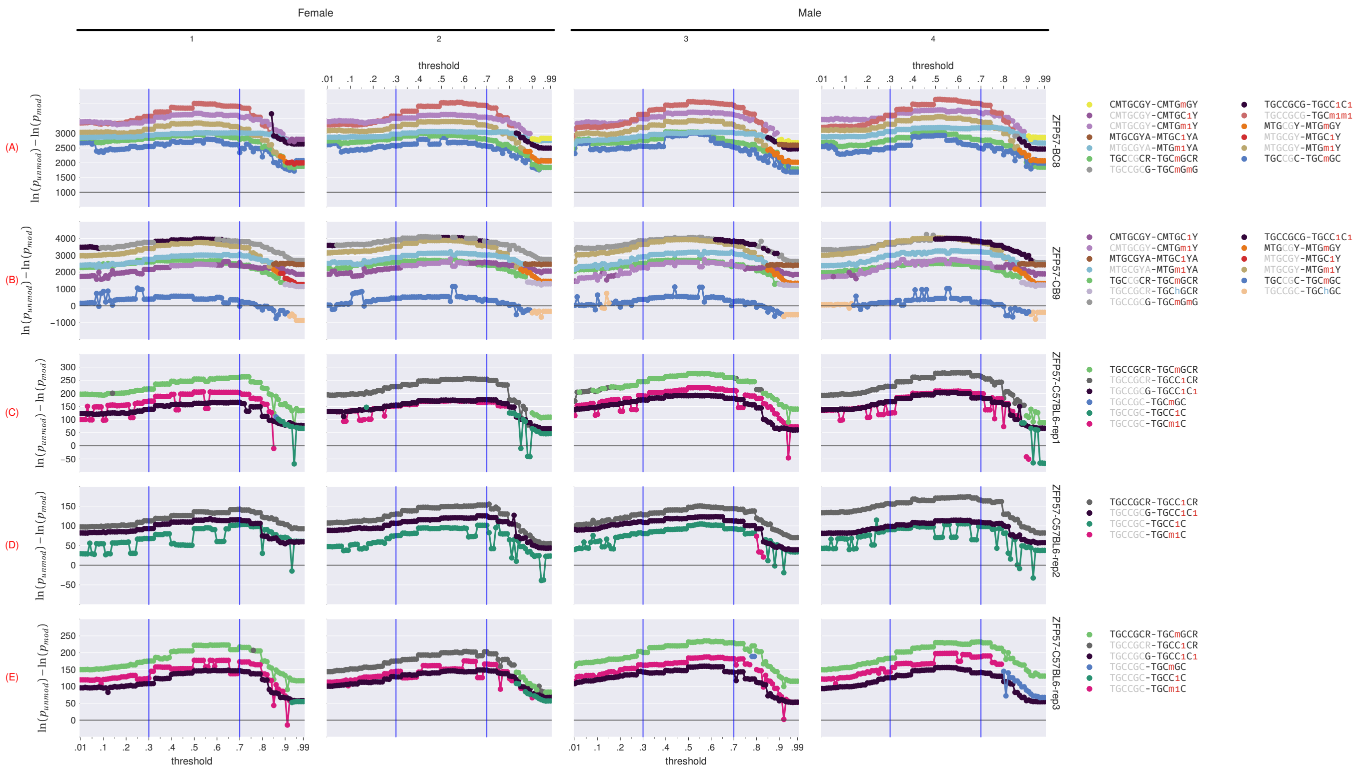

Fig. S3. Relationship between unmodified versus modified ZFP57 statistical significance of central enrichment (from CentriMo[55]) and modified base calling thresholds across different WGBS and oxWGBS specimens, in mice.[51] We compared each unmodified motif, at each threshold, to its top most significant modification. The displayed motif pairs changes at individual thresholds, depending on which motif pairs stay at the top. We called all ZFP57 peaks using the default MACS[62] threshold of $q = 0.05$. Sign of value indicates preference for the unmodified (negative) motif or the modified (positive) motif. Rows: single ChIP-seq replicates for a particular transcription factor target, consisting of all ZFP57 replicates (from Quenneville et al.[22], and BC8 and CB9 cells, from Strogantsev et al.[23]). Columns: replicates of WGBS and oxWGBS.

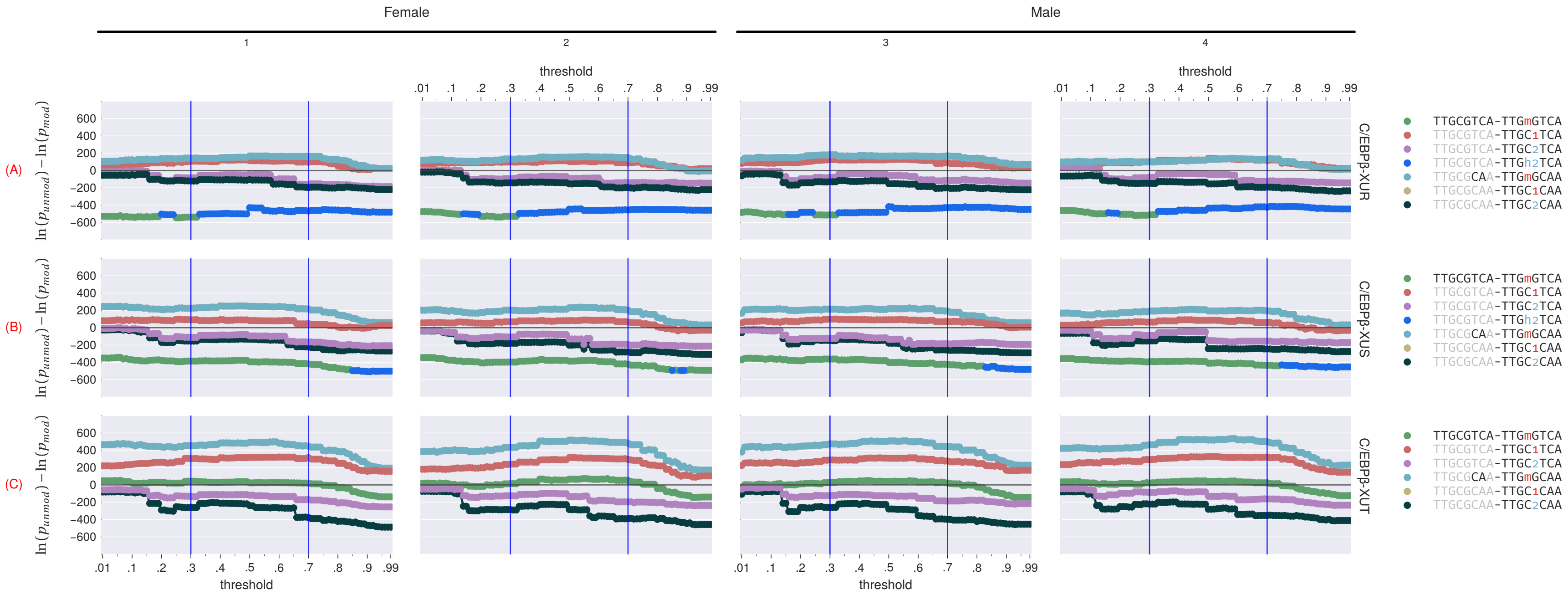

Fig. S4. Relationship between unmodified versus modified C/EBPβ statistical significance of central enrichment (from CentriMo[55]) and modified base calling thresholds across different WGBS and oxWGBS specimens, in mice.[51] We compared each unmodified motif, at each threshold, to its top three most significant modifications. The displayed motif pairs changes at individual thresholds, depending on which motif pairs stay in the top three. Sign of value indicates preference for the unmodified (negative) motif or the modified (positive) motif. Rows: single ChIP-seq replicates for a particular transcription factor target, consisting of all C/EBPβ replicates (`ENCFF001XUR`, `ENCFF001XUS`, and `ENCFF001XUT`). Columns: replicates of WGBS and oxWGBS. We additionally depict all of our other tested transcription factors in Fig. S2.

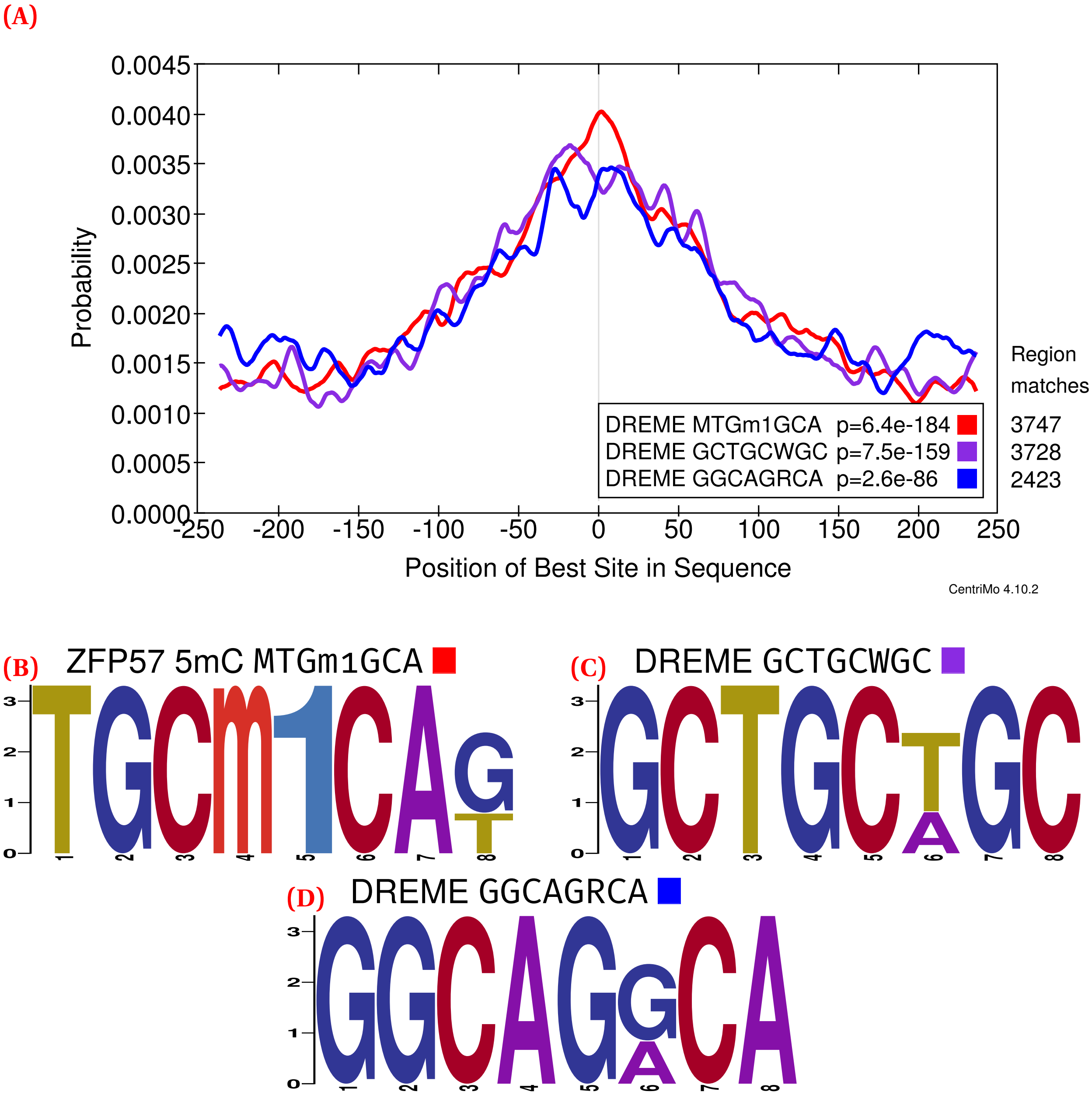

Fig. S5. ZFP57 (Strogantsev et al.[23] CB9; 56 142 ChIP-seq peaks) CentriMo analysis of *de novo* and JASPAR motifs (Methods). Listed p-values computed by CentriMo.[55] Depicts female replicate 2 of the combined WGBS and oxWGBS data[51] at a 0.7 modification threshold. (A) the CentriMo result with an expected ZFP57 methylated motif (red), top DREME unmodified motif (purple), and second DREME unmodified motif (blue). (B) Sequence logo of the reverse complement of the CentriMo result with an expected ZFP57 methylated motif. (C) Sequence logo of the top DREME unmodified motif. (D) Sequence logo of the second DREME unmodified motif.

(A)

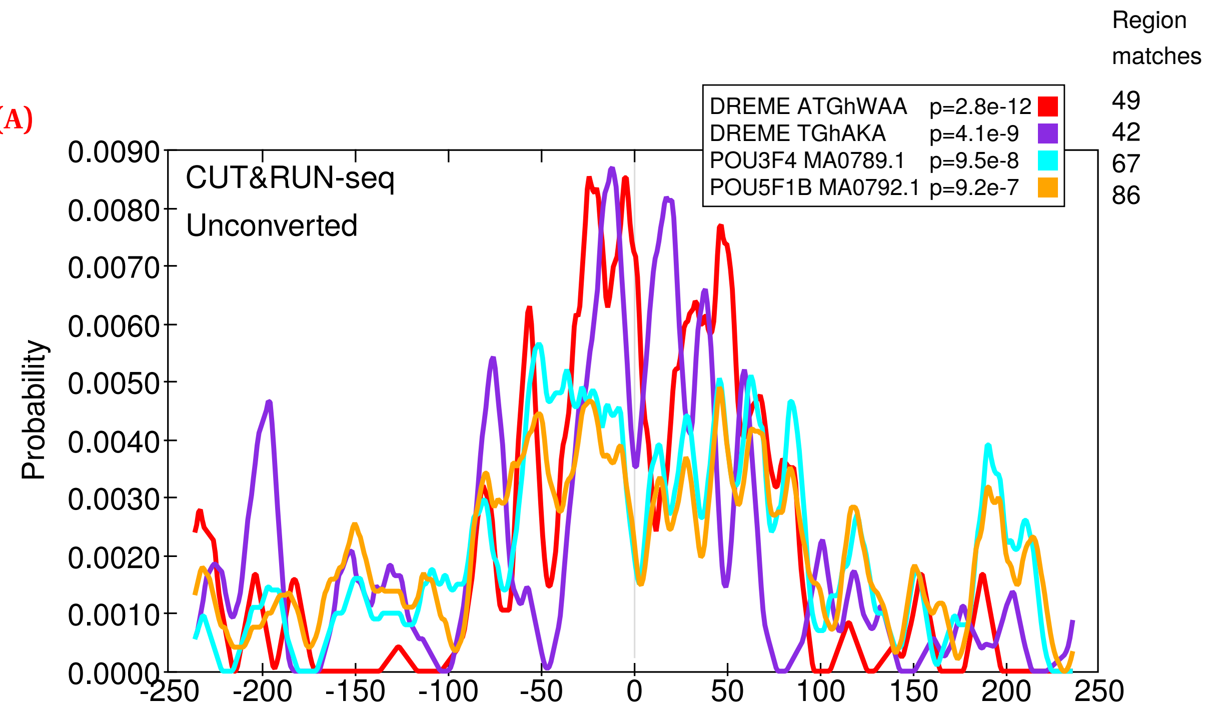
Region
matches
DREME ATGhWAA p=2.8e-12 49
DREME TGhAKA p=4.1e-9 42
POU3F4 MA0789.1 p=9.5e-8 67
POU5F1B MA0792.1 p=9.2e-7 86
CUT&RUN-seq
Unconverted
Probability
0.0090
0.0080
0.0070
0.0060
0.0050
0.0040
0.0030
0.0020
0.0010
0.0000
-250
-200
-150
-100
-50
0
50
100
150
200
250

(B)

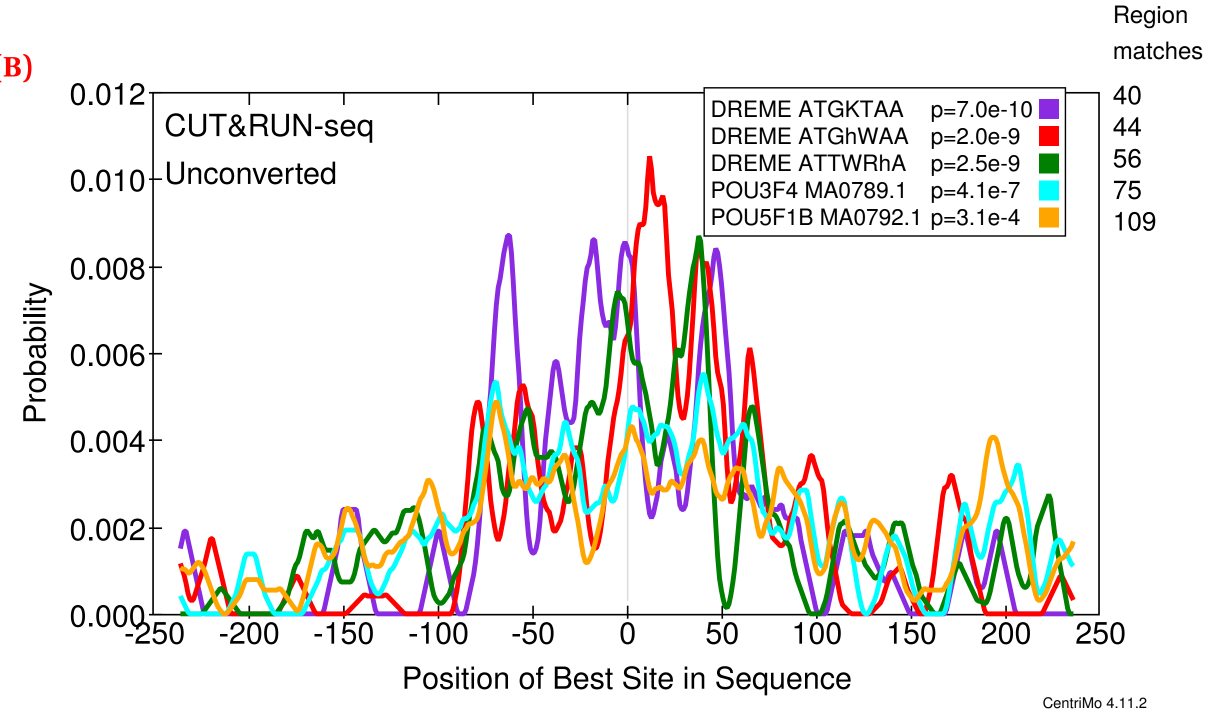
Region
matches
DREME ATGKTAA p=7.0e-10 40
DREME ATGhWAA p=2.0e-9 44
DREME ATTWRhA p=2.5e-9 56
POU3F4 MA0789.1 p=4.1e-7 75
POU5F1B MA0792.1 p=3.1e-4 109
CUT&RUN-seq
Unconverted
Probability
0.012
0.010
0.008
0.006
0.004
0.002
0.000
-250
-200
-150
-100
-50
0
50
100
150
200
250
Position of Best Site in Sequence
CentriMo 4.11.2

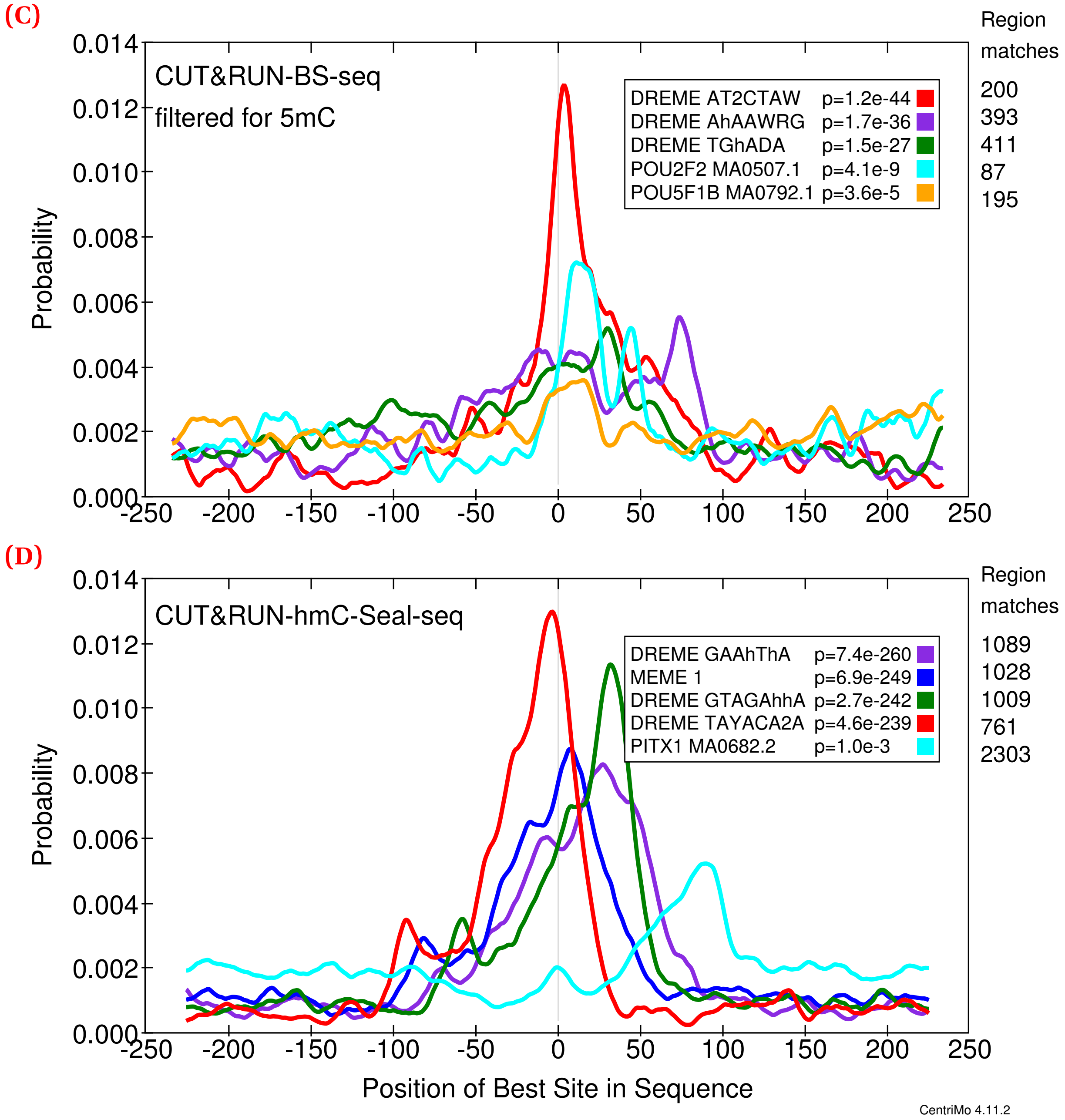

Fig. S6. CentriMo[55] results for OCT4 cleavage under targets and release using nuclease (CUT&RUN) in mouse embryonic stem cells (mESCs). Motifs include the top three DREME motifs with colour indicating rank: first (red); second (purple), third, where applicable (dark green). Motifs also include the top non-POU5F1 JASPAR motif (cyan), and the JASPAR POU5F1B motif (orange). Both of these motifs come from the JASPAR 2020[61] core vertebrate set. We generated these results using 500 bp regions centred upon the summits of MACS 2[62] peaks generated from those CUT&RUN fragments ≤ 120 bp. We called peaks using IgG controls and without any spike-in calibration (Methods). Listed p-values computed by CentriMo.[55] For consistency, we depict the JASPAR sequence logo using MEME's relative entropy calculation and colouring. Also depicts the top MEME[63] *de novo* motif (blue). We depict the first replicates of 5mC and 5hmC data in Fig. 5. **(A)** replicate 1 unconverted (227 CUT&RUN peaks) sequences. **(B)** replicate 2 unconverted (265 CUT&RUN peaks) sequences. **(C)** replicate 2 bisulfite-converted (methylated; 2077 CUT&RUN peaks) sequences. **(D)** replicate 2 hmC-Seal (11 615 CUT&RUN peaks) sequences.

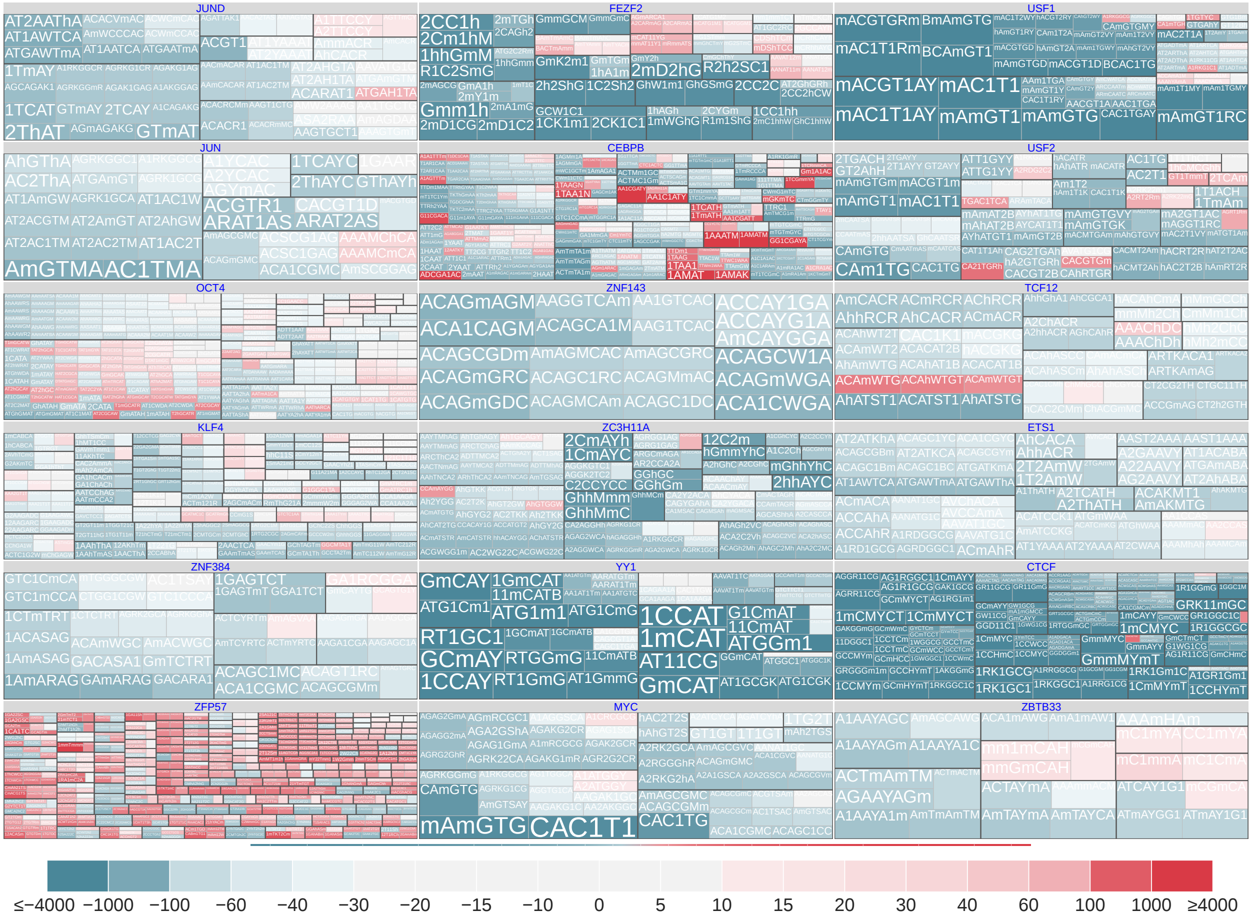

Fig. S7. Modified versus unmodified motifs, combining score and cluster information, for a wide array of transcription factors. These plots come from non–spike-in calibrated data, for the 500 bp regions surrounding peak summits. We clipped scores above or below ±4000 and plotted them at a threshold that maximizes dynamic range where most scores occur. Some combinations of the displayed hypothesis pairs have multiple data points. For example, these points can correspond to multiple identical hypothesis pairs, but for different data sub-types or stringencies. We aggregated these multiple data points by plotting the maximum score. An asymmetric, diverging, colour scale further highlights modification-preferring motifs.
