# Supplementary material for: Modeling methyl-sensitive transcription factor motifs with an expanded epigenetic alphabet
Source: Genome Biol. 2024 Jan 8;25:11. doi: 10.1186/s13059-023-03070-0 (PMC10773111; doi:10.1186/s13059-023-03070-0)
Supplement: Supplementary file 2 — Additional file 2: Appendix A. Recommendations for modified nucleobase nomenclature. Table S1. Recommendations for the nomenclature of modified nucleobases, grouped by the unmodified nucleobase. [file 13059_2023_3070_MOESM2_ESM.pdf]

Appendix A Recommendations for modified nucleobase nomenclature

Interest in different covalent DNA modifications and improvements in sequencing technologies have created a greater need for computational analyses of modified sequence data. To encourage standardization, we recommend symbols for various modified nucleobases (Table S1). We use lowercase letters (`a`–`z`) for specific nucleobase forms and numerals (`0`–`9`) to specify complements without any information loss. The list does not provide symbols for all DNA bases found in nature, but may provide guidance for those who need to select symbols for these bases.

The list here reserves specific symbols in an attempt to reduce contradictory definitions. We reserved all uppercase letters of the Latin alphabet for allocation by the International Union of Pure and Applied Chemistry (IUPAC), in addition to those already specified.[95] We use lowercase letters, and there do not exist sufficient unassigned letters to restrict ourselves to those without a different meaning in uppercase. Many applications, including the MEME Suite, only support Latin letters and numerals.

We recommend referring to any covalent modification at atomic position ⟨*position*⟩, specific modification ⟨*modification*⟩, and original nucleobase ⟨*base*⟩ as ⟨*position*⟩⟨*modification*⟩⟨*base*⟩. For example, this leads to "5mC" as the abbreviation for 5-methylcytosine. In particular, we recommend using no punctuation to demarcate the position from the modification and placing the numeral before the base modified. For example, others have occasionally abbreviated 6-methyladenine as "m6A", but we recommend the use of "6mA" instead when the modification occurs in DNA rather than in RNA.[178]

We incorporated the core symbols for cytosine modifications (Table 1) into our broader recommendations Table S1. While we specified a set of ambiguity codes for our usage here (Table 2), we do not recommend general definitions. Instead, we suggest reserving (1) the end of the lowercase Latin alphabet and (2) numerals. Avoiding universal assignment of these codes makes it more likely that the 62 symbols of the alphanumeric alphabet will prove sufficient for a variety of uses. As implemented here, we recommend assigning ambiguity codes starting from the end of the Latin alphabet or set of numerals, beginning with the most equivocal ambiguity code (such as `z` or `9`).

Nucleobase			Complement	
Abbreviation	Name	Symbol	Name	Symbol
Covalent modifications of cytosine				
5mC	5-methylcytosine	m	guanine:5mC	1
5hmC	5-hydroxymethylcytosine	h	guanine:5hmC	2
5fC	5-formylmethylcytosine	f	guanine:5fC	3
5caC	5-carboxylmethylcytosine	c	guanine:5caC	4
Covalent modifications of thymine				
5hmU	5-hydroxymethyluracil	g	adenine:5hmU	
5fU	5-formyluracil	e	adenine:5fU	
5caU	5-carboxyluracil	b	adenine:5caU	
Covalent modifications of adenine				
6mA	6-methyladenine	a	thymine:6mA	
Covalent modifications of guanine				
8-oxoG	8-oxoguanine	o	adenine:8-oxoG (mismatch)	
Reserved synthetic bases				
Xao	xanthosine	n		

Table S1. Recommendations for the nomenclature of modified nucleobases, grouped by the unmodified nucleobase. Numeral symbols for complements allow their differentiation from bases complementary to unmodified forms. One could reassign these numerals when analyzing other sets of covalent modifications. Since xanthosine can base-pair with multiple bases,[179] we list no complement for it.
