# Supplementary material for: Modeling methyl-sensitive transcription factor motifs with an expanded epigenetic alphabet
Source: Genome Biol. 2024 Jan 8;25:11. doi: 10.1186/s13059-023-03070-0 (PMC10773111; doi:10.1186/s13059-023-03070-0)
Supplement: Supplementary file 3 — Additional file 3. Review history. [file 13059_2023_3070_MOESM3_ESM.docx]

Review History

**First round of review**

**Reviewer 1**

**Were you able to assess all statistics in the manuscript, including the appropriateness of statistical tests used?**

Yes, and I have assessed the statistics in my report.

**Comments to author:**

Vein et al. expanded the standard A/C/G/T DNA alphabet to include cytosine modifications and developed methods to identify transcription factor binding sites in modified DNA. They confirmed the preference of ZFP57 and C/EBPβ for methylated motifs and the c-Myc for unmethylated E-box motifs. They used these known binding preferences to tun model parameters by using grid research. Besides, they discovered novel modified motifs for a wide array of transcription factors. Finally, they validated predicted binding preferences of OCT4 using CUT&RUN experiments across conventional, methylation-, and hydroxymethylation-enriched sequences. The idea of expanding DNA alphabet is novel and has great potential to benefit the community. However, the organization of the paper needs to be substantially improved.

Major:

1. Since the authors aim to introduce a method, they should have a figure to demonstrate the workflow of their method.

2. Related to the first comment, it is hard to follow what kind of method they developed for identifying transcription factor binding sites since there is no such workflow. As far as I told from the manuscripts, they developed Cytomod to expand the DNA alphabet using cytosine modification data but using published tools such as MEME to identify the motif and CentriMo to quantify the preferences. In this case, the author should clarify their method and how it contributes to identifying the transcription factor binding site in modified DNA.

3. The authors put 'Expanded-alphabet genomes facilitate the analysis of modified base data' as their first result but need figures to demonstrate how the expanded-alphabet genomes facilitate the analysis and to what degree. The authors should explain it in a more quantified way. For example, comparing the identified motifs between the expanded-alphabet genome and the original genome and checking how many new motifs were found. And which chromosome benefited the most from this expanded-alphabet etc.

4. Related to comment 3, the authors could show some performance evaluations of the Cytomod in this section.

5. P5Line13, the author should explain the term 'threshold'. The author should also mention what other model parameters they tuned in this section and show the grid search results of those parameters if they existed since they mentioned tuning the model parameters in P6Line27-28.

6. P6Line60-61: More detailed instruction on how to use their method is needed. The GitHub page has no instructions on how to use Cytomod and how to combine it with MEME::Alphabet. The author should give a detailed manual on their GitHub page.

7. Figures 3, 4 can be combined to show the comparison more straightforwardly.

8. The information delivered in Figure 5 could be easier to follow. For example, the author could give some quantification panels in Figure 5 based on Tables 1 and 3 to directly show the information they want to deliver.

Minor:

1. Figures should be ordered by their appearance order in the context. For example, Fig. 2 is mentioned after Fig. 3. Thus, the order of these two figures needs to be switched.

2. Fig. 1 could be the supplementary figs instead of the first figure.

3. The author should explain the 'a variety of modified based calling threshold' and 'calibrated modified genome' in P4Line46 as all these terms jump from nowhere and have no reference to any method.

4. legend of Fig. 3A and Fig. 4A should move to the upper right corner.

5. P6Line12, the authors should give the method reference to 'score'.

6. As the authors mentioned Grau et al emphasized their model goes beyond PWMs. Comparing results between the expanded alphabet on conventional PWM and Grau's method would be interesting.

**Reviewer 2**

**Were you able to assess all statistics in the manuscript, including the appropriateness of statistical tests used?**

Yes, and I have assessed the statistics in my report.

**Comments to author:**

Epigenetic modifications have wide-spread roles in chromatin regulation. Currently we only encode DNA in a roughly 4 character alphabet (16 including degenerate nucleotides). DNA methylation is a covalent, heritable modification that has have been shown to attenuate transcription factor-DNA interactions. Here, Viner et al., proposed and expanded DNA alphabet to include modifications (namely, methylation). They demonstrate that this alphabet enables improved modeling of transcription factor recognition preferences. Overall, I think this paper is of general interest to the gene regulatory community and is technically sound. I expect that this type of expanded alphabet will be widely implemented in current and future DNA analysis tools.

I only have a few minor comments:

1) I find the writing a little clunky and could be trimmed down extensively.

2) The section "Our methods yield suitable base-calling thresholds..." seems out of order. It took me a couple of passes through the text and figure to understand the analysis. This might be more appropriate for supplementary materials, as it deals with the specifics of methylation calls.

3) The heatmap representations in Figure 5 do not clearly convey the desired information. If I understand correctly, the authors are demonstrating that certain TFs are methyl-sensitive. I might just shown the PWMs/logos for the top and bottom 10 or so motifs.

4) DNNs (deep neural nets) that attempt to predict epigenetic features from sequence are increasingly common. Currently, the one-hot encoding is the de facto standard for CNNs. This paper would benefit from discussing how to incorporate these extra "nucleotides" into a feature encoding or tokenization strategy.

**Authors Response**

**Point-by-point responses to the reviewers’ comments:**

Reviewer #1: Vein et al. expanded the standard A/C/G/T DNA alphabet to include cytosine modifications and developed methods to identify transcription factor binding sites in modified DNA. They confirmed the preference of ZFP57 and C/EBPβ for methylated motifs and the c-Myc for unmethylated E-box motifs. They used these known binding preferences to tun model parameters by using grid research. Besides, they discovered novel modified motifs for a wide array of transcription factors. Finally, they validated predicted binding preferences of OCT4 using CUT&RUN experiments across conventional, methylation-, and hydroxymethylation-enriched sequences. The idea of expanding DNA alphabet is novel and has great potential to benefit the community. However, the organization of the paper needs to be substantially improved.

*Response: We appreciate your review of this paper, your kind remarks, and important points for us to clarify. You have highlighted an important difficulty in our work: because of its size and complexity, we have found it challenging to organize it to be most easily understood by a variety of audiences. We have had to make some compromises in these regards, though we hope the organizational changes here have improved its clarity. We previously worked with a professional editor to improve the organization of the text, and hope that we were now more successful in conveying things clearly.*

Major:

1. Since the authors aim to introduce a method, they should have a figure to demonstrate the workflow of their method.

*Response: We appreciate this useful suggestion and have now done so. Please find this figure in Methods, as Figure 6.*

2. Related to the first comment, it is hard to follow what kind of method they developed for identifying transcription factor binding sites since there is no such workflow. As far as I told from the manuscripts, they developed Cytomod to expand the DNA alphabet using cytosine modification data but using published tools such as MEME to identify the motif and CentriMo to quantify the preferences. In this case, the author should clarify their method and how it contributes to identifying the transcription factor binding site in modified DNA.

*Response: Thank you for highlighting this important point. Figure 6, added in response to your previous comment, should help.*

*We would also like to clarify that our extensive additions to the MEME Suite were completed with some of the software’s authors. Indeed, our second-last author is the main senior developer and co-PI behind the MEME Suite.*

*We have added to Results:*

*These modified sequences have a central role not just in our method, but also in enabling bioinformatic analyses of modifications more generally (Discussion).*

*In addition to creating new standalone software to instantiate our expanded alphabet concept, we also updated the MEME Suite and associated software, implementing the ability to work with custom alphabets, such as our expanded epigenomic alphabet.*

*We have also added to Discussion:*

*Prior to our introduction of the expanded epigenetic alphabet concept, it was not possible to perform direct, non-subsequent analyses to assess modified bases like 5mC in motifs that leverage standard algorithms (such as for motif elucidation) or existing engineering (such as comprehensive analysis pipelines). While some of the transcription factors examined had previously known preferences for modified or unmodified cytosines, the motifs found here are, in effect, completely new, due to this expanded alphabet allowing the joint consideration of cytosine modification status and multiple-base sequence specificity.*

*Without the expansion of the alphabet, one cannot directly use motifs to search for nor distinguish between any unmodified or modified bases. As such, our analyses and motifs result directly from this expansion and all our Results demonstrate quantified elucidation of expanded alphabet motifs. One can see this most emphatically in our validation results, where we show that we can directly detect motifs pertaining to bound chromatin fractions specifically containing the predicted modification of interest (Figure 5).*

3. The authors put 'Expanded-alphabet genomes facilitate the analysis of modified base data' as their first result but need figures to demonstrate how the expanded-alphabet genomes facilitate the analysis and to what degree. The authors should explain it in a more quantified way. For example, comparing the identified motifs between the expanded-alphabet genome and the original genome and checking how many new motifs were found. And which chromosome benefited the most from this expanded-alphabet etc.

*Response: We appreciate this suggestion, which has let us provide increased clarity here, as described below.*

*We have added additional detail and quantification in Results:*

*Overall, we assessed a total of 144 distinct transcription factors, across all datasets. Grouping these by modification preference, with each factor potentially in multiple groups to reflect the possibility of mixed preferences, we found:*

*● 71 transcription factors appeared to bind only to unmodified motifs. Each transcription factor in this grouping had no positively-scoring motifs.*

*● 14 transcription factors appeared to bind predominantly to modified motifs. Each transcription factor in this grouping had motifs with an upper quartile score ≥0.*

*● 9 transcription factors had no clear modified motif preferences. Each transcription factor in this grouping had no motifs with a score outside of [−50, 50], excepting those transcription factors where every motif scored positively. Of these transcription factors, 5 had three or fewer significant motifs.*

*We have also added related comments to Discussion in response to comment 2, above.*

4. Related to comment 3, the authors could show some performance evaluations of the Cytomod in this section.

*Response: We have added to Methods:*

*Cytomod performance*

*Generally, one needs to run Cytomod only once per analysis project, so we did not focus on improving runtime performance. For the analyses of hundreds of ChIP-seq datasets undertaken here, outside of development, initial tests, and threshold calibration, we only had to run Cytomod four times. In other words, we only needed four modified genome sequences to complete most of our work, two per each selected threshold, one for the mouse genome and one for the human genome. Since future work will likely not require further Cytomod development and threshold calibration, others using Cytomod would not need to run it more than a few times either. Nonetheless, Cytomod performs quickly already, considering each run produces a complete modified genome assembly.*

*Typically, Cytomod completes work on an ∼3 Gbp genome in considerably less than 8 h on a single core of an Intel Xeon E5-2650 v2 2.6 GHz Linux workstation, and it uses less than 24 GB of RAM. Cytomod requires output disk storage space of approximately the same size as the unmodified input assembly. We benchmarked some specific illustrative runtimes. First, creating a modified GRCh38/hg38 assembly with 1 modification track (x; WGBS data only) took Cytomod 3 h 15 min 26 s. Second, creating a modified NCBI m37/mm9 assembly with 4 modification tracks (m, h, x, z; both WGBS and oxWGBS data) took 4 h 14 min 40 s.*

5. P5Line13, the author should explain the term 'threshold'. The author should also mention what other model parameters they tuned in this section and show the grid search results of those parameters if they existed since they mentioned tuning the model parameters in P6Line27-28.

*Response: Thank you for highlighting this issue.*

*We have added to Results: We constructed expanded-alphabet modified genomes, making discrete calls from the continuous output of our modification calling pipeline. The pipeline output numbers in [0, 1] indicating the strength of evidence for a modification at each position. We determined whether to call a base modified or not by comparing the output values to a threshold value fixed across the whole genome (Methods).*

6. P6Line60-61: More detailed instruction on how to use their method is needed. The GitHub page has no instructions on how to use Cytomod and how to combine it with MEME::Alphabet. The author should give a detailed manual on their GitHub page.

*Response: Thank you for highlighting the lack of clarity in regard to our software. We have updated Cytomod’s* [*GitHub page*](https://github.com/hoffmangroup/cytomod) *to provide usage information.*

*The MEME::Alphabet Perl module is not related to creation of expanded alphabets nor Cytomod, but rather serves as a module to teach various other Perl modules or scripts how to handle our expanded-alphabet sequences. So users do not directly use this module, but we provide it to help other package authors make use of our alphabet, as we did in the MEME Suite.*

*We have updated in Results:*

*In addition to creating new standalone software to instantiate our expanded alphabet concept, we also updated the MEME Suite and associated software, implementing the ability to work with custom alphabets… This Perl module does not create expanded alphabets or expanded alphabet genome sequences, but rather provides capabilities for other Perl software to read and handle biomolecular sequences with expanded alphabets. Moreover, it provides a reference for implementing the same capabilities in other programming languages.*

7. Figures 3, 4 can be combined to show the comparison more straightforwardly.

*Response: We appreciate this suggestion, as it highlighted a lack of clarity in our exposition of these figures: these two figures are not meant to be compared with each other. Rather, they are designed to both show, at distinct thresholds, that our methods are capable of finding the expected results in our controls. Thus, they remain separate, as comparisons between them would not be meaningful. We have added to Results:*

*We show the assessments at different thresholds not for comparing against each other, but to demonstrate the robustness of our results to varying the threshold. At both the minimum and the maximum of our modified base calling threshold calibration, we can elucidate expected modified motif preferences. In both cases, the expected motif has strong central enrichment across ChIP-seq peaks. Only the central region of these motif enrichment analyses are relevant.*

8. The information delivered in Figure 5 could be easier to follow. For example, the author could give some quantification panels in Figure 5 based on Tables 1 and 3 to directly show the information they want to deliver.

*Response: We appreciate this excellent suggestion. By considering the nature of our figure, our overall goals in what we wanted to depict, and similar comments by Reviewer 2, we decided to make improvements to this figure (now Figure 4).*

*We have added additional panels, one below JUN and another below OCT4, that depict the most, middle, and least modification-preferring motif’s sequence logos. We then show where these key motifs are in the treemap and further show their positions on the colour scale, indicating their score. This serves as an additional quantification panel that, we hope, also clarifies the information we are trying to deliver in this figure.*

Minor:

1. Figures should be ordered by their appearance order in the context. For example, Fig. 2 is mentioned after Fig. 3. Thus, the order of these two figures needs to be switched.

*Response: Thank you for catching this—we swapped the order of these figures.*

2. Fig. 1 could be the supplementary figs instead of the first figure.

*Response: We agree. This figure is now Figure S1.*

3. The author should explain the 'a variety of modified based calling threshold' and 'calibrated modified genome' in P4Line46 as all these terms jump from nowhere and have no reference to any method.

*Response: We apologize for these oversights and have now added additional cross-references to Results: We elaborate upon these base calling thresholds and their use in creating calibrated genomes in the next subsection and in Methods.*

4. legend of Fig. 3A and Fig. 4A should move to the upper right corner.

*Response: While these legends may appear to be blocking data, they actually do not. CentriMo plots for most conventional datasets, such as those in these figures, are only relevant at their central portions. Additionally, in these figures, as with most conventional CentriMo plots, the lines are exactly symmetrical WRT the other side, barring minor numerical deviations. In this figure, the lines shown on the left are a mirror image of the lines on the right. CentriMo defaults to this legend position, as one is not meant to focus on this irrelevant plot area. We only moved it in other plots, when we found that there could have been slight deviations from this symmetry, or portions of the central peaks we wished to highlight, due to the novel types of data we apply CentriMo to in this work. We have added to Results:*

*Only the central region of these motif enrichment analyses are relevant.*

5. P6Line12, the authors should give the method reference to 'score'.

*Response: We added a cross-reference to Methods.*

6. As the authors mentioned Grau et al emphasized their model goes beyond PWMs. Comparing results between the expanded alphabet on conventional PWM and Grau's method would be interesting.

*Response: Thank you for this interesting suggestion. Both our works use PWMs, while Grau et al.’s methods also allow extension of TFFMs. These two models have been quite extensively compared, to some degree in their own work, but also in the unmodified context in the original* [*TFFM paper by Mathelier and Wasserman*](https://journals.plos.org/ploscompbiol/article?id=10.1371/journal.pcbi.1003214)*. In our view, outside modification-specific sensitivity, there is not much to be added by a comparison, beyond those already performed in these papers and others.*

*We agree that a comparison of modification sensitivity is interesting and that TFFMs could potentially improve upon our work. As we noted, their work was not able to find C/EBPβ’s preference for methylated motifs, whereas ours not only immediately did so, but also sensitively found its converse preference for hydroxymethylated motifs. This is a key finding in this area, and indeed why we first used it as a control. As such, we do not believe that a comparison with Grau et al.’s models would yield any non-trivial results. Thus, we would prefer not to include such a comparison rather than further extend the focus of the paper.*

*Note that in spite of C/EBPβ serving as a control, we did not need to tune our method in any way to correctly obtain this result: as we show in Figure 3 and Figure S4, we were always able to reliably determine this, within a wide range of thresholds. Any detailed comparison would accordingly be both beyond the scope of our present work, but also best left until other methods are able to reliably demonstrate valid preferences for well-known TFBSs.*

Reviewer #2:

Epigenetic modifications have wide-spread roles in chromatin regulation. Currently we only encode DNA in a roughly 4 character alphabet (16 including degenerate nucleotides). DNA methylation is a covalent, heritable modification that has have been shown to attenuate transcription factor-DNA interactions. Here, Viner et al., proposed and expanded DNA alphabet to include modifications (namely, methylation). They demonstrate that this alphabet enables improved modeling of transcription factor recognition preferences. Overall, I think this paper is of general interest to the gene regulatory community and is technically sound. I expect that this type of expanded alphabet will be widely implemented in current and future DNA analysis tools.

*Response: We greatly appreciate your review of this paper, your kind remarks, and important points for us to clarify. We address your specific comments below.*

I only have a few minor comments:

1) I find the writing a little clunky and could be trimmed down extensively.

*Response: [below response is same as earlier text in response to Reviewer 1] You have highlighted an important difficulty in our work: because of its size and complexity, we have found it challenging to organize it to be most easily understood by a variety of audiences. We have had to make some compromises in these regards, though we hope the organizational changes here have improved its clarity. We previously worked with a professional editor to improve the organization of the text, and hope that we were now more successful in conveying things clearly.*

2) The section "Our methods yield suitable base-calling thresholds..." seems out of order. It took me a couple of passes through the text and figure to understand the analysis. This might be more appropriate for supplementary materials, as it deals with the specifics of methylation calls.

*Response: We appreciate your highlighting a potential point of confusion for many readers. We have added additional cross-references and expository text, which we hope will serve to clarify this issue.*

3) The heatmap representations in Figure 5 do not clearly convey the desired information. If I understand correctly, the authors are demonstrating that certain TFs are methyl-sensitive. I might just shown the PWMs/logos for the top and bottom 10 or so motifs.

*Response: We appreciate this excellent suggestion. By considering the nature of our figure, our overall goals in what we wanted to depict, and similar comments by Reviewer 1, we decided to make improvements to this figure (now Figure 4). We have now added additional panels, one below JUN and another below OCT4, that depict the most, middle, and least modification-preferring motif’s sequence logos. We then show where these key motifs are in the treemap and further show their positions on the colour scale, indicating their score. This serves as an additional quantification panel that, we hope, also clarifies the information we are trying to deliver in this figure.*

4) DNNs (deep neural nets) that attempt to predict epigenetic features from sequence are increasingly common. Currently, the one-hot encoding is the de facto standard for CNNs. This paper would benefit from discussing how to incorporate these extra "nucleotides" into a feature encoding or tokenization strategy.

*Response: We agree that this is an exciting and rapidly developing area. Our work might one day serve as a basis for such a neural network approach incorporating epigenetic modifications, and we appreciate your bringing this to our attention. We have added to Discussion some advice for potential encodings in the context of building expanded-alphabet neural networks:*

*For representation of sequence data with modifications as input to neural network architectures, one could use our expanded alphabet. One could use this for either modified motif elucidation or for overall classification of a transcription factor’s propensity to bind modified bases. Naively, one could encode the expanded epigenetic alphabet by simply extending the standard one-hot DNA encoding, as long used in motif elucidation, 90 to add our additional symbols. Without additional changes, however, we would not recommend this approach because of the substantial disparity in modified base frequencies.*

*For representing expanded epigenetic alphabet data, we would suggest using an approach where significantly lower frequency of modified bases compared to unmodified has a lower impact. One might instead encode all nucleobases as vectors representing their functional groups, as recently done in a similar unmodified context. 91 Alternatively, one might adapt recent work which designed filters to create sparse codes, similar to images, that can effectively encode DNA motifs. 92 This work constructs the encoding from PWMs, as we do for unmodified bases. It uses a one-hot encoding for DNA, but should allow for the ncorporation of altered background frequencies. These approaches likely have more resilience to biases in this expanded alphabet context, while still enabling the ready application of modern neural networks to modified nucleobase data.*

*90. Workman, C. T. & Stormo, G. D. ANN-SPEC: a method for discovering transcription factor binding sites with improved specificity in Pacific symposium on biocomputing (eds Altman, R. B., Lauderdale, K., Dunker, A. K., Hunter, L. & Klein, T. E.) (2000), 464–75.*

*91. Pap, G., Zoltán, G., Ádám, K., Tóth, L. & Hegedüs, Z. Transcription factor binding site detection using convolutional neural networks with a functional group-based data representation. Journal of Physics: Conference Series 1824, 012001 (2021).*

*92. Chu, S. K. & Stormo, G. D. Finding motifs using DNA images derived from sparse representations. Bioinformatics 39:6 (ed Martelli, P. L.) btad378 (2023).*

**Second round of review**

**Reviewer 1**

The authors have now addressed my concerns.

**Reviewer 2**

The authors have addressed my concerns/comments satisfactorily.
